# Expression of early growth responsive gene-1 in the visual cortex of monocular form deprivation amblyopic kittens

**DOI:** 10.1186/s12886-021-02161-5

**Published:** 2021-11-15

**Authors:** Haobo Fan, Ying Wang, Xiuping Tang, Liyuan Yang, Weiqi Song, Yunchun Zou

**Affiliations:** 1 Department of Ophthalmology, Affiliated Hospital of North Sichuan Medical College, Nanchong, China; 2 Department of Optometry, North Sichuan Medical College, Nanchong, China; 3 Innovative Platform for Basic Medicine, North Sichuan Medical College, Nanchong, China

**Keywords:** Early growth responsive gene-1 (Egr-1), Amblyopia, Form deprivation, Visual cortex, Kitten

## Abstract

**Purpose:**

The present study compared the expression of early growth responsive gene-1 (Egr-1) in visual cortex between amblyopia kittens and normal kittens, and to explore the role of Egr-1 in the pathogenesis of amblyopia.

**Methods:**

A total of 20 healthy kittens were randomly divided into deprivation group and control group with 10 kittens in each group. Raised in natural light, and covered the right eye of the deprived kittens with a black opaque covering cloth. Pattern visual evoked potentials (PVEP) were measured before and at the 1st, 3rd and 5th week after covering in all kittens. After the last PVEP test, all kittens were killed. The expression of Egr-1 in the visual cortex of the two groups was compared by immunohistochemistry and in situ hybridization.

**Results:**

PVEP detection showed that at the age of 6 and 8 weeks, the P100 wave latency in the right eye of deprivation group was higher than that in the left eye of deprivation group (*P* < 0.05) and the right eye of control group (*P* < 0.05), while the amplitude decreased (*P* < 0.05). The number of positive cells (*P* < 0.05) and mean optical density (*P* < 0.05) of Egr-1 protein expression in visual cortex of 8-week-old deprivation group were lower than those of normal group, as well as the number (*P* < 0.05) and mean optical density of Egr-1 mRNA-positive cells (*P* < 0.05).

**Conclusions:**

Monocular form deprivation amblyopia can lead to the decrease of Egr-1 protein and mRNA expression in visual cortex, and then promote the occurrence and development of amblyopia.

## Background

Amblyopia is one of the important diseases causing visual loss of children in the world, and its incidence rate in Asia is 1.09% [1]. In recent years, with the in-depth research on the pathogenesis of amblyopia in molecular biology, neurobiology and other disciplines [2], relevant studies have confirmed that the basic basis of amblyopia treatment lies in the existence of visual plasticity during the sensitive period of visual development [3]. The plasticity mechanism of visual development is related to many neurotransmitters, but the specific pathogenesis of amblyopia has not been fully elucidated, and the changes of critical period and plasticity of amblyopia can not be explained in detail at the molecular level [4–6].

Synapse is currently considered as the most critical link in amblyopia, and its plasticity can be divided into long-term potentiation (LTP) and long-term depression (LTD) according to time [7, 8]. As a member of the Egr family of Immediate-early genes (IEGs), early growth responsive gene-1 (Egr-1) acts as a transcription factor encoding zinc finger structure. The increase of Egr-1 expression is associated with synaptic plasticity, memory consolidation, LTP induction and learning [9, 10]. At the same time, immediate early genes also have the effect of coupling short-term signals with long-term changes [11]. However, there has been no study on the correlation between the expression of Egr-1 and amblyopia. Therefore, we examined changes in Egr-1 in the visual cortex in amblyopia to investigate the significance of this body in the pathogenesis of amblyopia and provide theoretical support for the occurrence and development of amblyopia.

## Methods

### Animals

We used 20 healthy 3-week-old kittens weighing between 240 g and 350 g, regardless of coat color and gender. The examination reveals no opacity of refractive medium or obvious abnormality of fundus, and the refractive error was + 1.25 ~ + 3.25D. All kittens were kept in a room with plenty of natural light and a temperature of 24 ± 1 °C. Up to the age of 5 weeks, all kittens were fed milk powder and water 6 times a day as they were unable to feed on their own. After 5 weeks of age, the kitten has fed solid food and drank water four times a day. The kittens were provided by the Experimental Animal Center of North Sichuan Medical College. The study was supervised by the Experimental Animal Ethics Committee of North Sichuan Medical College and it has been performed according to the ARRIVE guidelines (NSMC Appl. No. 2021 [24]).

### Animals model establishment

**Fig. 1:**
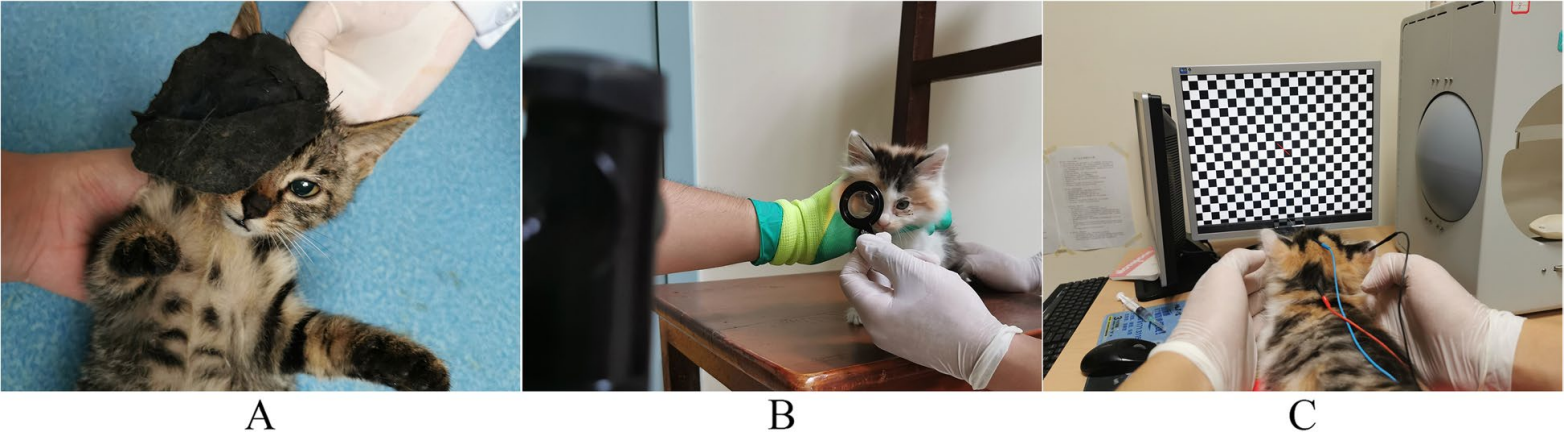
Establishment and examination of animal model. **A** Cover the right eye of the kitten with a covering cloth. **B** Detection the diopter of the kitten with a band ophthalmoscope C: Schematic diagram of PVEP detection

The Kittens were divided into a control group (*n* = 10) and a monocular deprivation group (n = 10) by random number table methods. The right eye of all deprived kittens was covered with black opaque eye mask to ensure the formation of amblyopia. At the 1st, 3rd and 5th week after covering, the Pattern Visual Evoked Potential (PVEP) test was used to measure each eye of kittens in the control group and the monocular deprivation group. According to the American Veterinary Medical Association (AVMA) Guidelines for the Euthanasia of Animals (2020), we euthanized all kittens with 2% sodium pentobarbital (100 mg/kg) after the last PVEP test. The visual cortex was separated and the expression of Egr-1 was detected by immunohistochemistry and in situ hybridization. (Fig. 1).

### PVEP detection

The Kittens were intraperitoneally injected with 1 % pentobarbital sodium (35 mg/kg). The refractive error of the kittens was detected by static retinoscopy and corrected accordingly with lenses. During the PVEP test on all kittens, three animal electrode needles (RL-1223000030-RC-D, Roland Consult Stasche Finger Gmbh) were inserted respectively in the following ways: the blue positive pole inserted the middle of the forehead, the red negative pole inserted the middle of the occiput, and the black ground pole inserted the subcutaneous back of the ear tip in the same direction as the measured eye. Position the kitten on a platform 40 cm away from the display screen and position the head so that the center of the rear pole of the retina is level with the center of the computer screen. PVEP (RETI-port/scan 21, Roland Consult Stasche Finger Gmbh) measurement parameters were adjusted to checkerboard flip stimulation, 0.3 cpd mode, and the time frequency was 1 Hz, a contrast of 97%, sampling time of 300 ms and superimposed 64 times. Measurements of each eye were repeated three times to obtain an average. (Fig. 1).

### Immunohistochemical detection

Paraffin sections were dewaxed to water and placed in 3% hydrogen peroxide solution and phosphate buffer saline (pH 7.4) (Boster Biological Technology Co., Ltd., China, AR0030) in turn to block endogenous peroxidase. The slices were placed in a repair box containing citric acid (pH 6.0) (Boster Biological Technology Co., Ltd., China, AR0024) antigen repair buffer for antigen repair. The tissue was then evenly covered with 5% BSA blocking solution in the culture dish for serum blocking. Followed by the addition of the first antibody (Egr-1) (Beijing Biosynthesis Biotechnology Co., Ltd., China, BS-1076R), second antibody (Biotin Conjugated goat anti-rabbit IgG) and strept avidin biotin complex (SABC) (Boster Biological Technology Co., Ltd., China, SA1022). DAB (Boster Biological Technology Co., Ltd., China, AR1022) was used to show colour, and positive results ranged from yellow to brownish yellow. The nucleus of haematoxylin staining (Beijing Solarbio Science & Technology Co., Ltd., China, Ltd., G1080) was blue and sealed via dehydration. Chemiluminescence was measured using image analysis by Image-Pro Plus.

### In situ hybridization

Paraffin sections were dewaxed in water and boiled in repair solution for 10 min. After natural cooling, digested with protease K (20 μl/ml) at 37 °C for 30 min. Then 3% methanol-hydrogen peroxide was added, and the slide was placed in phosphate buffer saline (pH 7.4) (Boster Biological Technology Co., Ltd., China, AR0033) to block endogenous peroxidase. After pre-hybridization, Egr-1 mRNA probe (5`-GAGGA GATGA TGCTG CTGAG CAACG GGGCT-3`; 5`-GCCTT TGCCA CTCAG TCGGG CTCCC AGGAG-3`; 5`-CCTTT TCTCC CAGCA CAATT GAAAT TTGCT-3`) hybridization solution containing the probe was added (Boster Biological Technology Co., Ltd., China, MK1748) at concentration of 20 μl. Hybridization was conducted at 37 °C in an incubator overnight, and then the hybridization solution was washed away. BSA blocking solution was then added, followed by a drop of mouse anti-digoxigenin-labelled peroxidase (Boster Biological Technology Co., Ltd., China, MK1748). DAB (Boster Biological Technology Co., Ltd., China, AR1022) was used to show colour, and positive results ranged from yellow to brownish yellow. The nucleus of haematoxylin staining (Beijing Solarbio Science & Technology Co., Ltd., China, Ltd., G1080) was blue and sealed via dehydration. Chemiluminescence was measured using image analysis by Image-Pro Plus.

### Statistical analysis

SPSS 25.0 statistical software was used. The data are expressed as means ± standard deviation ($\overline{X}$± *s*), using one-way analysis of variance (SNK), paired-samples *t*-test and two independent sample *t*-test. The P100 waves of each eye in the control group and the deprivation group were analyzed by one-way ANOVA (SNK). The comparison between the right eye and left eye of the deprivation group was performed by paired-samples *t*-test, and the comparison between the control group and the deprivation group was performed by two independent sample *t*-test. The results of immunohistochemistry and in situ hybridization were analyzed by independent samples *t*-test. The correlation among PVEP data and the protein and mRNA expression from the control and deprivation groups was performed by Person correlation coefficient.

## Results

### P100 wave of PVEP

**Fig. 2:**
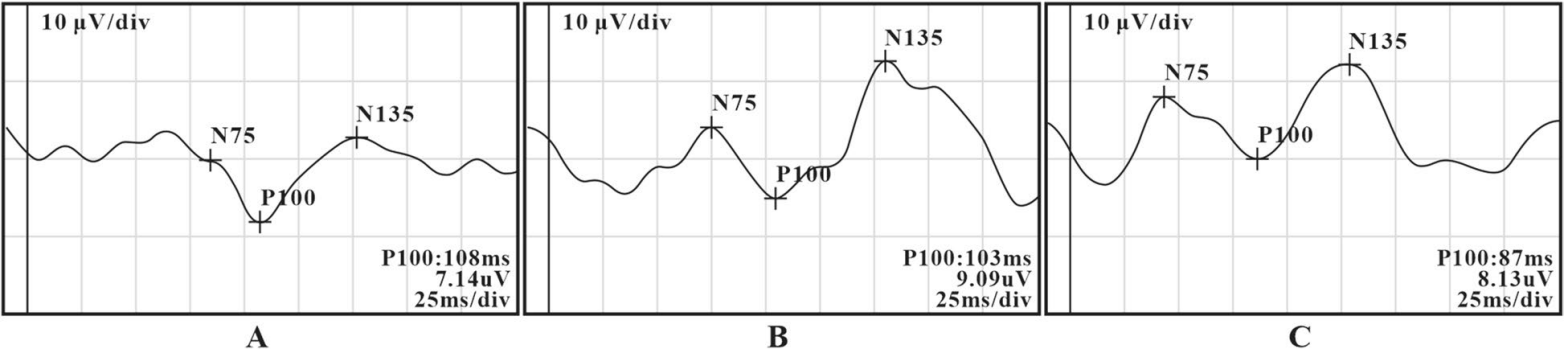
PVEP waveform of kittens in each group after covering for 5 weeks. **A** Right eye in deprivation group; **B** Left eye in deprivation group; **C** Right eye of control group

The PVEP examination showed that the main wave image of kitten PVEP showed N75-P100-N135 complex wave, which was composed of two positive waves and one negative wave, showing an “M” shape (Fig. 2). At the age of 3 weeks, there was no statistical difference in latency (*F* = 0.254, *P* = 0.778) and amplitude (*F* = 0.009, *P* = 0.990) of P100 wave between right eye and left eye of deprivation group and right eye of control group.

At the age of 6 weeks, the latency (*F* = 26.768, *P* < 0.001) and amplitude (*F* = 12.142, *P* < 0.001) of P100 wave were statistically different among the three groups; the latency of P100 wave in the right eye of deprivation group was higher than that in the left eye of deprivation group (*P* = 0.0002) and the right eye of control group (*P* = 0.0004). The amplitude of the right eye of the deprivation group was lower than that of the left eye of the deprivation group (*P* < 0.001) and the right eye of the control group (*P* < 0.0001).

At the age of 8 weeks, the latency of P100 wave in the right eye of deprivation group was higher than that in the left eye of deprivation group (*P* < 0.0001) and the right eye of control group (*P* < 0.0001). The amplitude of the right eye of the deprivation group was lower than that of the left eye of the deprivation group (*P* = 0.0001) and the right eye of the control group (*P* = 0.0001).

**Fig. 3:**
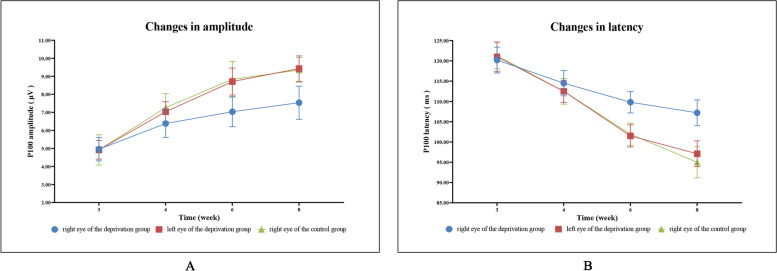
The latency and amplitude trend of P100 waves in 3-week-old to 8-week-old kittens. With increasing age, the amplitude of the P100 wave in the each eye of the control group and the deprivation group gradually increased **A**, and the latency shortened gradually **B**

Therefore, at the age of 6 weeks, monocular form deprivation amblyopia has formed in the right eye of deprived kittens (Fig. 3, Tables 1and 2). (relevant data is available at https://figshare.com/s/b237d9430f77928923df).

**Table 1:** P100 amplitude in each group. ($\overline{X}$±*S*, μV)

Time (weeks)	right eye of the deprivation group	left eye of the deprivation group	right eye of the control group	*F*	*P*
3	4.958 ± 0.646	4.923 ± 0.515	4.917 ± 0.839	0.009	0.990
4	6.383 ± 0.771	7.039 ± 0.771	7.266 ± 0.778^a^	3.749	0.037
6	7.036 ± 0.834	8.712 ± 0.748^a^	8.834 ± 0.995^a^	12.142	< 0.001
8	7.538 ± 0.921	9.430 ± 0.705^a^	9.354 ± 0.691^a^	17.008	< 0.001
*F*	17.634	88.407	51.491		
*P*	< 0.001	< 0.001	< 0.001		

^a^Compared with the right eye of the deprivation group, there was a difference (*P* < 0.05)

**Table 2:** P100 latency in each group. ($\overline{X}$±*S*, ms)

Time (weeks)	right eye of the deprivation group	left eye of the deprivation group	right eye of the control group	*F*	*P*
3	120.20 ± 3.19	121.00 ± 3.66	121.30 ± 3.29	0.254	0.778
4	114.50 ± 3.11	112.50 ± 2.73	112.50 ± 3.17	1.326	0.282
6	109.80 ± 2.64	101.50 ± 2.73^a^	101.90 ± 2.77^a^	26.768	< 0.001
8	107.20 ± 3.19	97.10 ± 3.18^a^	95.00 ± 3.85^a^	32.772	< 0.001
*F*	31.827	109.499	111.537		
*P*	< 0.001	< 0.001	< 0.001		

^a^Compared with the right eye of the deprivation group, there was a difference (*P* < 0.05)

### Immunohistochemical

**Fig. 4:**
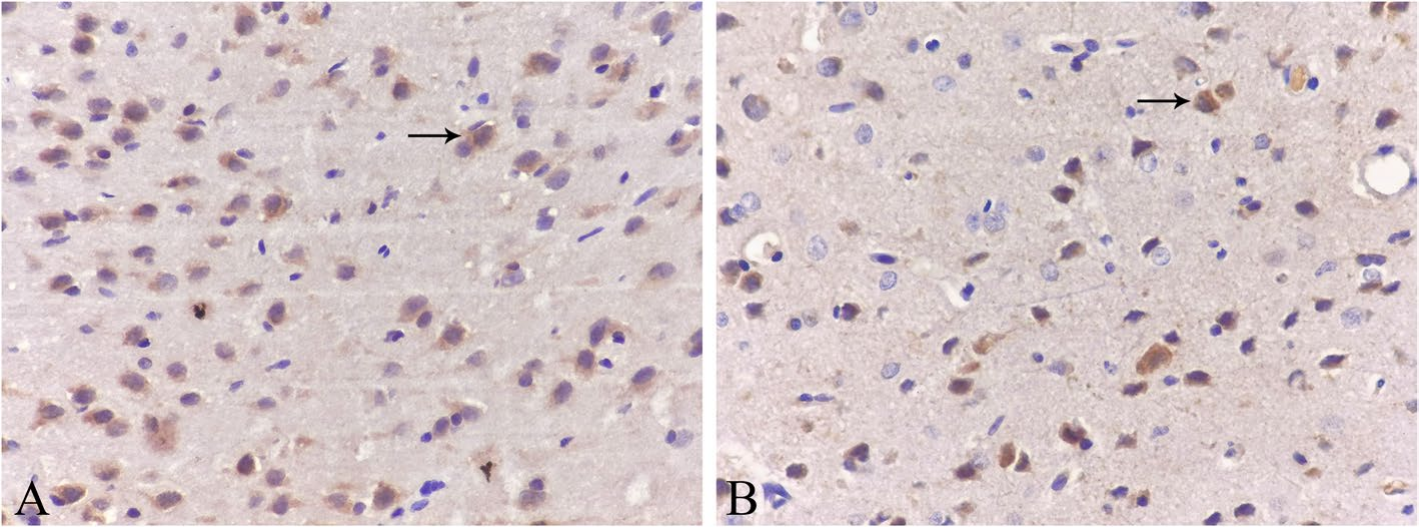
Immunohistochemical performance in visual cortex in each group (DAB X400). The black arrow in the picture indicates positive cells. The protein of Egr-1 positive expression in the cytoplasm of neurons was brown-yellow. At the age of 8 weeks, there were more positive cells in the control group **A** and fewer in the deprivation group **B**

Four visual fields were randomly selected from each slice for statistical analysis. All of the sections showed Egr-1 protein expression in the cytoplasm, which was brown-yellow, and the nucleus was blue. At 8 weeks of age, the mean optical density of positive cells in the control group was higher than that in the deprivation group (*P* < 0.001). The number of positive cells in the control group was greater than that in the deprivation group (*P* < 0.001) (Fig. 4, Table 3). (relevant data is available at https://figshare.com/s/b237d9430f77928923df).

**Table 3:** Egr-1 immunohistochemistry of visual cortex of 8-weeks-old kittens

Group	Mean optical density of positive	Positive cell number
Control group	0.014365 ± 0.005558	51.28 ± 15.99
Deprivation group	0.006403 ± 0.002859	31.85 ± 14.14
*t*	−8.057	−5.754
*P*	< 0.001	< 0.001

### In situ hybridization

**Fig. 5:**
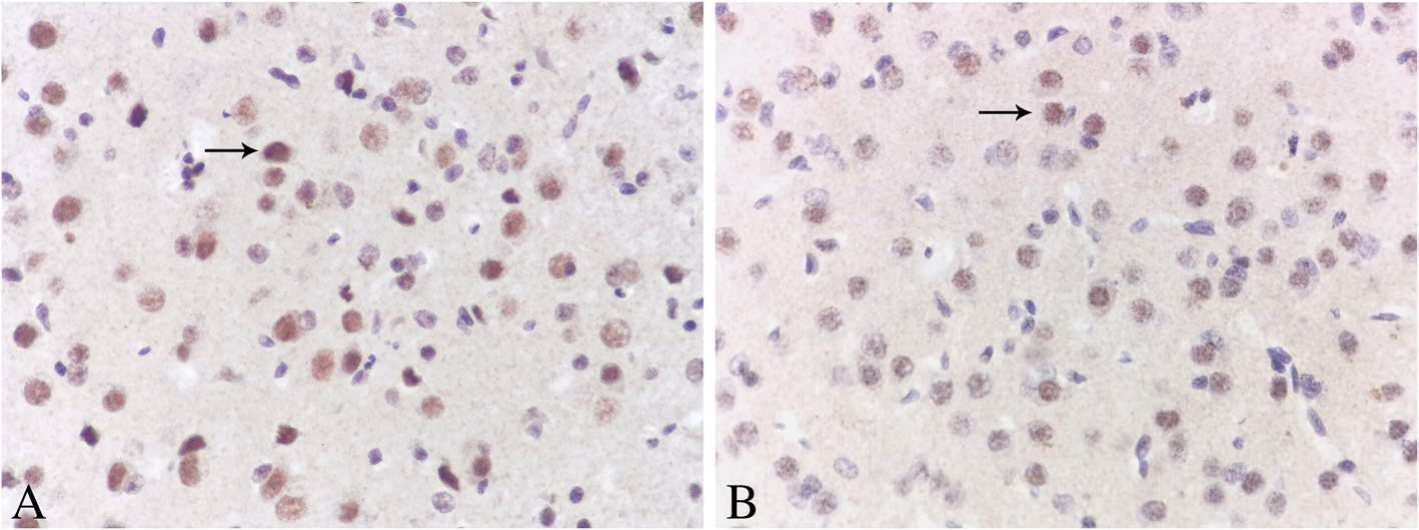
In situ hybridization performance in visual cortex in each group (DAB X400). The black arrow in the picture indicates positive cells. The mRNA of Egr-1 positive expression in the nucleus of neurons was brown-yellow. At the age of 8 weeks, there were more positive cells in the control group **A** and fewer in the deprivation group **B**

Four visual fields were randomly selected from each slice for statistical analysis. All of the sections showed Egr-1 mRNA expression in the nucleus, which was brown-yellow, and the nucleus was blue. At 8 weeks of age, the mean optical density of positive cells in the control group was higher than that in the deprivation group (*P* < 0.001). The number of positive cells in the control group was greater than that in the deprivation group (*P* < 0.001) (Fig. 5, Table 4). (relevant data is available at https://figshare.com/s/b237d9430f77928923df).

**Table 4:** Egr-1 in situ hybridization of visual cortex of 8-weeks-old kittens

Group	Mean optical density of positive	Positive cell number
Control group	0.022897 ± 0.004059	80.58 ± 11.25
Deprivation group	0.015012 ± 0.005543	66.88 ± 14.62
*t*	−7.258	−4.697
*P*	< 0.001	< 0.001

### The analysis of correlation

Person correlation coefficient analysis showed that the amplitude of PVEP was positively correlated with mean optical density of positive and positive cell number from Egr-1 protein and mRNA expression. And the latency of PVEP was negatively correlated with with mean optical density of positive and positive cell number from Egr-1 protein and mRNA expression. (Table 5).

**Table 5:** The analysis of correlation among PVEP and the protein or mRNA expression. (*PCCs*, *P*)

Group	PVEP		IHC		ISH	
	amplitude	latency	Mean optical density of positive	Positive cell number	Mean optical density of positive	Positive cell number
PVEP amplitude	–	−0.565, 0.009	0.705, 0.0005	0.675, 0.001	0.474, 0.0346	0.682, 0.0009
PVEP Latency	−0.565, 0.009	–	−0.739, 0.0001	− 0.61, 0.004	−0.733, 0.0002	− 0.668, 0.0013

## Discussion

At present, the classic method of modeling form deprivation amblyopia is upper eyelid suture [12–14]. In the past, our team selected 3-week-old kittens to establish amblyopia models by monocular form deprivation and used the Suture Covering Method [15], that is, using black covering cloth instead of eyelid suture to model amblyopia. The continuous observation of PVEP changes in experimental animals was realized by suture while preventing eye injury caused by eyelid suture. This method can effectively block the light from covering the eyes of animals in all directions and successfully establish amblyopia animal models. However, during the modeling period, some kittens still had head movements, which led to light passing through the side of the covering cloth. In order to improve this situation, this experiment increased the diameter of the covering cloth in the original covering method, and made a black opaque covering ring with a certain height in the center of the covering cloth to further enhance the covering efficiency.

Some studies have shown that the dark environment will affect the plasticity of visual cortex in kittens, which will increase the amplitude of P100 waves and improve their eyesight [16, 17]. Therefore, in the process of establishing amblyopia kittens model, we only removed the black opaque covering cloth during detection. Moreover, we use natural light throughout the whole process to ensure the stability of their biological rhythm.

In this experiment, checkerboard flip stimulation was used to detect PVEP, and the wave images of PVEP in kittens were recorded, which were composed of two positive waves and one negative wave, showing an “M” shape. By comparing the amplitude and latency of P100 waves between different eyes at the same time and the same eye at different times, we found that P100 waves in three groups of PVEP detection, with the increase of age, the latency gradually shortened and the amplitude gradually increased. The amplitude of P100 wave decreased and the latency of P100 wave increased in the right eye of deprivation group compared with the left eye of deprivation group and the right eye of control group. This is similar to the previous amblyopia modeling results by other schemes [18, 19]. The critical period of visual development in kittens is from 4 weeks to 16 weeks after birth [20, 21]. During this period, abnormal visual environment may lead to the influence of visual development. In the PVEP test, with the increase of age, the change of amplitude and latency of P100 wave in amblyopia eyes decreased compared with normal eyes. The changes of PVEP suggest that the appearance of form deprivation amblyopia in kittens makes various fine visual functions of visual system such as analytical perception, motion perception and stereo vision change. At present, some studies believe that establishing binocular stereo vision is one of the purposes of preventing and treating amblyopia [22]. The realization of this goal is based on synaptic plasticity.

Many neurotransmitters are considered to be related to the critical period of amblyopia and its plasticity changes [23, 24]. Moreover, in some studies, some proto-oncogenes are closely related to it [25, 26]. As a class of proto-oncogenes encoding transcription factors, immediate early genes have the effect of coupling short-term signals with long-term changes, mainly including C-fos, Egr family and Arc, among which many transcription factors are regulated by visual activities. Among them, there is sufficient evidence to show the association between C-fos and amblyopia [13, 27]. The transcription factor of Egr-1 in the Egr family of immediate early genes is essential in the changes of visual cortex plasticity [28]. Egr-1 is a transcription factor encoding zinc finger structure, and its expression increases during synaptic plasticity, memory consolidation, LTP induction and learning [7, 8]. Cytoskeleton related gene Arc, as one of the target genes of Egr-1, is an effector molecule induced by synaptic activity and plays an important role in late LTP. Studies have shown that under certain conditions, Egr-1 can regulate the transcription of late activity-dependent Arc gene in the hippocampus CA1 region, and the immediate early gene Arc can connect the change pattern of neural activity and synaptic plasticity because of its pluripotent and fine tuning system, thus optimizing the information storage of nervous system [28]. In addition, previous studies on amblyopic animal models have found that there are structural changes in ganglion cells, lateral geniculate body and visual cortex of amblyopic animals, accompanied by a decrease in synaptic density, which will lead to further changes in their functions [29, 30].

Immunohistochemistry and in situ hybridization were used to compare and analyze the expression of Egr-1 protein and mRNA in the visual cortex of 8-week-old amblyopia kittens and normal kittens. We found that positive cell numbers and the mean optical density of positive in the visual cortex of 8-week-old amblyopic kittens and normal kittens decreased. And the results show that there is a correlation among PVEP, IHC and ISH data. The appearance of this result provides strong proof for the association between Egr-1 and amblyopia. However, in terms of PVEP, although the amplitude of PVEP in the left eye of deprived kittens was lower than that in the right eye of the control group, there was no statistical difference between the two groups. Similar phenomena have been found in other studies [31]. Considering that the optic chiasma of cats is semi-chiasma, the change of visual cortex will affect both eyes. We speculate that this may be due to PVEP reflecting the conduction, transmission and excitation intensity of ocular ganglion cells to occipital visual cortex. Studies have shown that amblyopia is related to macular development and retinal fibrous layer thickness in addition to the changes of visual cortex-related protein expression [32–34].

The occurrence of amblyopia is related to synaptic plasticity changes, and long-term changes will occur in the process of amblyopia. The balance of excitation and depression at axonal level of the visual cortex is the condition of maintaining normal development and function of the visual cortex, and it is also an important factor affecting the plasticity of visual system [35]. The plasticity of nerve is mainly manifested in the plasticity of synaptic structure and function [36, 37]. Egr-1 and Arc genes are closely related to long-term changes of synapses. At present, most studies also show that perceptual learning can improve the visual function of amblyopia patients [5]. Perceptual learning can effectively restore the visual function through repeated visual stimulation and visual experience with supervision and feedback [38–40]. Researchers found that its mechanism may be related to the balance of excitement and depression related to plasticity, and Egr-1 and Arc play an important role in learning and memory of humans and animals [41, 42].

This study not only confirmed the correlation between Egr-1 protein and mRNA expression in visual cortex and amblyopia, that is to say, due to the unequal input of binocular vision, the number and morphology of visual cortex cells may change, resulting in the decrease of Egr-1 protein and mRNA expression, which further affects the normal physiological function of visual cortex and affects visual development, thus promoting the occurrence and development of amblyopia. But also indirectly verified the correlation between perceptual learning and amblyopia.

However, this study still has some limitations. In the process of modeling, we only use the random number table method to divide the kittens into two groups, but did not randomize the deprived eyes of the deprived kittens. On the other hand, we proved that the expression protein and mRNA of Egr-1 was down-regulated in the visual cortex of amblyopia kittens, but we failed to detect dynamically change of Egr-1, or set different groups to observe its changes with age.

## Conclusions

To sum up, the animal model of amblyopia can be established and the dynamic measurement of eyeball parameters can be realized by using the method of ocular covering. Based on this method, the expression of Egr-1 in the visual cortex decreased significantly in amblyopia animal model. This experiment speculates that Egr-1 plays an important role in visual development. This study provides a new idea and direction for further exploring how monocular form deprivation regulates visual cortex neurons, and for treating amblyopia and deeply understanding its pathogenesis.

## Data Availability

The datasets used and analysed during the current study are available from the corresponding author on reasonable request. Or all relevant datasets related to the study can be found in the specified (database.https://figshare.com/s/b237d9430f77928923df).

## Abbreviations

Egr-1: Early growth responsive gene-1
LTP: Long-term potentiation
LTD: Long-term depression
IEGs: Immediate-early genes
PVEP: Pattern Visual Evoked Potential

## References

[1] Fu Z, Hong H, Su Z (2020). Global prevalence of amblyopia and disease burden projections through 2040: a systematic review and meta-analysis. Brit J Ophthalmol.

[2] Santhan G, Jai K, Aditya K, Abhishek P (2019). Simplified updates on the pathophysiology and recent developments in the treatment of amblyopia: A review. Indian J Ophthalmol.

[3] Holmes JM, Levi DM (2018). Treatment of amblyopia as a function of age. Vis Neurosci.

[4] Castaldi E, Lunghi C, Morrone MC (2020). Neuroplasticity in adult human visual cortex. Neurosci Biobehav R.

[5] Eleni P, Ioannis A, Gail M (2019). The treatment of amblyopia: current practice and emerging trends. Graefes Arch Clin Exp Ophthalmol.

[6] Hensch TK, Quinlan EM (2018). Critical periods in amblyopia. Vis Neurosci.

[7] John L, Katherine C, Megha S, Alcino JS (2018). Memory formation depends on both synapse-specific modifications of synaptic strength and cell-specific increases in excitability. Nat Neurosci.

[8] Alberini CM (2009). Transcription factors in long-term memory and synaptic plasticity. Physiol Rev.

[9] Baroncelli L, Lunghi C (2021). Neuroplasticity of the visual cortex: in sickness and in health. Exp Neurol.

[10] Nicholas AH, Brittany FO, Lauren AM (2018). Differential expression of the immediate early genes c-Fos , Arc , Egr-1 , and Npas4 during long-term memory formation in the context preexposure facilitation effect (CPFE). Neurobiol Learn Mem.

[11] Teissier A, Le Magueresse C, Olusakin J (2020). Early-life stress impairs postnatal oligodendrogenesis and adult emotional behaviour through activity-dependent mechanisms. Mol Psychiatry.

[12] ARAKAWA K, PEACHEY NS, CELESIA GG, RUBBOLI G (1993). Component-specific effects of physostigmine on the cat visual evoked potential. Exp Brain Res.

[13] Zhu T, Ma C, Liu A (2013). Research on acupuncture regulation of visual cortex c-Fos protein expression in monocular deprivation cats. J Acupunct Tuina Sci.

[14] van Sluyters RC (1978). Recovery from Monocular Stimulus Deprivation Amblyopia in the Kitten. Elsevier.

[15] Li B, Zou Y, Yin X, Tang X, Fan H. Expression of brain-derived neurotrophic factor in the lateral geniculate body of monocular form deprivation amblyopic kittens. Eur J Ophthalmol. 2020:1120672120953341. 10.1177/1120672120953341. Epub ahead of print. 10.1177/1120672120953341 32873060

[16] Montey KL, Quinlan EM (2011). Recovery from chronic monocular deprivation following reactivation of thalamocortical plasticity by dark exposure. Nat Commun.

[17] Mitchell DE, MacNeill K, Crowder NA (2016). Recovery of visual functions in amblyopic animals following brief exposure to total darkness. J Physiol.

[18] Burns BD, Pritchard R (1968). Cortical conditions for fused binocular vision. J Physiol.

[19] Regina HFDM, Stefania A, Bruna B (2013). Visual evoked potential importance in the complex mechanism of amblyopia. Int Ophthalmol.

[20] Baro JA, Lehmkuhle S, Kratz KE (1990). Electroretinograms and visual evoked potentials in long-term monocularly deprived cats. Invest Ophthalmol Vis Sci.

[21] Duffy KR, Lingley AJ, Holman KD, Mitchell DE (2016). Susceptibility to monocular deprivation following immersion in darkness either late into or beyond the critical period. J Comp Neurol.

[22] Levi DM, Knill DC, Bavelier D (2015). Stereopsis and amblyopia: a mini-review. Vis Res.

[23] Adema R, Thomas B, Hirofumi M (2019). Emerging roles of synapse organizers in the regulation of critical periods. Neural Plast.

[24] Frank S (2014). Plasticity of the Visual Cortex and Treatment of Amblyopia. Curr Biol.

[25] Nikolaienko O, Patil S, Eriksen MS, Bramham CR (2018). Arc protein: a flexible hub for synaptic plasticity and cognition. Semin Cell Dev Biol.

[26] Ruth EC, Jeremy MH (2018). Transcriptional and post-translational regulation of arc in synaptic plasticity. Semin Cell Dev Biol.

[27] Yu M (2014). Relationship between monocularly deprivation and amblyopia rats and visual system development. Asian Pac J Trop Med.

[28] Zsuzsa P, Carine C, Serge L (2011). Contribution of Egr1/zif268 to activity-dependent arc/Arg3.1 transcription in the dentate gyrus and area CA1 of the Hippocampus. Front Behav Neurosci.

[29] Mitchell DE, Sengpiel F (2009). Neural Mechanisms of Recovery following Early Visual Deprivation. Philosophical Transactions: Biological Sciences.

[30] Lynne K (2019). Understanding the development of amblyopia using macaque monkey models. Proc Natl Acad Sci.

[31] Li B, Zou Y, Li L (2019). Therapeutic effect of vasoactive intestinal peptide on form-deprived amblyopic kittens. BMC Ophthalmol.

[32] Al-Haddad CE, El MG, Mahfoud ZR (2013). Macular ultrastructural features in amblyopia using high-definition optical coherence tomography. Br J Ophthalmol.

[33] Song WK, Lee SC, Lee ES (2010). Macular thickness variations with sex, age, and axial length in healthy subjects: a spectral domain-optical coherence tomography study. Invest Ophthalmol Vis Sci.

[34] Al-Haddad C, Mehanna CJ, Ismail K (2018). High-definition optical coherence tomography of the macula in Deprivational amblyopia. Ophthalmic Surg Lasers Imaging Retina.

[35] Daphne B, Levi DM, Li RW (2010). Removing brakes on adult brain plasticity: from molecular to behavioral interventions. J Neurosci.

[36] Linnea RV, Patric KS (2017). Synaptic Plasticity, Metaplasticity and Depression. Curr Neuropharmacol.

[37] Evanthia N, William AC (2018). Calcium Channels, Synaptic Plasticity, and Neuropsychiatric Disease. Neuron.

[38] Levi DM, Li RW (2009). Improving the Performance of the Amblyopic Visual System. Philos Trans Biol Sci.

[39] Astle AT, PV MG, Webb BS (2011). Can Human Amblyopia be Treated in Adulthood?. Strabismus.

[40] Pineles SL, Aakalu VK, Hutchinson AK (2020). Binocular Treatment of Amblyopia: A Report by the American Academy of Ophthalmology. Ophthalmology.

[41] Zsuzsa P, Elise M, Alexandra V (2014). Zif268/Egr1 gain of function facilitates hippocampal synaptic plasticity and long-term spatial recognition memory. Philos Trans R Soc B Biol Sci.

[42] Isabelle S, Samme V, Chris H (2018). Transient and localized optogenetic activation of somatostatin-interneurons in mouse visual cortex abolishes long-term cortical plasticity due to vision loss. Brain Struct Funct.

